# Numerical Simulation Study on the Corrosion Behaviour of Q345 Steel in a Simulated Marine Thermocline

**DOI:** 10.3390/ma17153808

**Published:** 2024-08-01

**Authors:** Jiezhen Hu, Junhao Zeng, Wenjuan Liu, Peichang Deng, Xin Hu, Peilin Wang

**Affiliations:** 1 College of Mechanical Engineering, Guangdong Ocean University, Zhanjiang 524088, China; hujz@gdou.edu.cn (J.H.); 21122050142@stu.gdou.edu.cn (J.Z.); 2112005019@stu.gdou.edu.cn (W.L.); 2112205051@stu.gdou.edu.cn (X.H.); 2112205053@stu.gdou.edu.cn (P.W.); 2 Zhanjiang Key Laboratory of Corrosion and Protection of Ocean Engineering Equipment, Zhanjiang 524088, China; 3 Guangdong Provincial Ocean Equipment and Manufacturing Engineering Technology Research Center, Zhanjiang 524088, China; 4 College of Chemistry and Environment, Guangdong Ocean University, Zhanjiang 524088, China

**Keywords:** marine thermocline, corrosion behaviour, Q345 steel, numerical simulation, galvanic corrosion

## Abstract

Changes in temperature, pH, dissolved oxygen content, and nutrients, which are key factors that cause metal corrosion, are common in marine thermoclines. To study the corrosion behaviours and reveal the corrosion mechanisms of metals in a marine thermocline, COMSOL 6.2 software is used in this paper. With this software, the corrosion behaviour of Q345 steel in a thermocline is numerically simulated, and a simulated marine thermocline is built indoors for experimental research purposes. The corrosion behaviour and mechanism of Q345 steel in a marine thermocline were investigated through numerical simulation, electrochemical testing, and corrosion morphology observation. After 21 days of immersion in the simulated marine thermocline, Q345 steel specimens at different depths are shown to have undergone vertical galvanic corrosion, with two anodes and two cathodes. At depths of 70 m and 150 m, the Q345 steel becomes the anode in the galvanic corrosion reaction, while at depths of 110 m and 190 m, the Q345 steel becomes the cathode in the galvanic corrosion reaction. The cathode is protected by the anode and has a relatively low corrosion rate. The main reason underlying these phenomena is that there are large differences in the dissolved oxygen contents and temperatures at different depths in a thermocline. The different dissolved oxygen contents lead to differences in the oxygen concentrations of Q345 steel specimens at various depths. These variations trigger galvanic coupling corrosion. Moreover, the difference in temperature further aggravates the degree of galvanic corrosion.

## 1. Introduction

A marine environment is a very complex corrosive environment that is usually divided into five zones in the vertical direction: the marine atmosphere zone, wave splash zone, tidal difference zone, seawater fully immersed zone, and sea mud zone [1,2]. Different types of marine engineering equipment are in the various zones of this environment. The corrosion behaviours of these systems differ due to the changing service environments in different zones. To date, global researchers investigating the corrosion behaviours and mechanisms of metal materials in marine environments have focused mainly on the marine atmosphere zone, surface shallow sea zone, and deep-sea zone. In the marine atmosphere zone, Zhao et al. [3] studied the atmospheric corrosion behaviour and mechanism of a 7A85 aluminium alloy. The experimental results showed that the mechanical properties of the bare 75A2 aluminium alloy deteriorate significantly in the marine atmosphere, mainly because the metal forms a thin electrolyte liquid film on the surface, which promotes metal corrosion. By comparing the corrosion patterns of metals in the water surface zone, tidal zone, and total immersion zone after submerging samples for 1–2 years, Mao et al. and Fozan et al. [4,5] concluded that the corrosion rates of metals are the highest in the oceanic tidal zone, and significant waterline corrosion occurs, proving that the corrosion environment in this region is the harshest [6,7]. For the study of the submerged zone of seawater, Jeffery [8] investigated the corrosion of vertical mild steel in seawater; the experimental results showed that the intensity of metal corrosion in the submerged zone depends on the length of the sample below the water line to a certain extent, and the longer the sample is, the greater the attenuation. Cai [9] investigated the hydrogen seepage and stress corrosion cracking behaviours of AISI4135 high-strength steels in the marine submerged zone.

Numerical simulation is the process of realizing some real-world functions and characteristics by constructing real-world conditions in the simulation model and using computers to conduct virtual tests on these geometric models [10]. Because numerical simulations are based on certain theoretical analyses and experimental research, they can be used to identify the corrosion-prone components of complex structures, to effectively predict the depth of corrosion, and to intuitively reveal the corrosion mechanisms of metals in a relatively short period; moreover, numerical simulations are favoured by researchers in the field of metal corrosion research [11,12,13,14,15,16,17]. Xie et al. [18] demonstrated that it is feasible to simulate corrosion defects with different inclinations in underwater pipelines using finite element simulation. Hu [19] used Maxwell simulation 16.0 software to simulate and study the corrosion and thinning characteristics of water-cooled wall pipes. Chauhan [20] used a concrete damage plasticity model to study the damage behaviour of reinforced concrete, obtained numerical predictions of the damage pattern, and verified the applicability of the model with experimental results. COMSOL Multiphysics is a multi-physics simulation software based on the finite element method, which allows users to simulate real-world physical phenomena by solving Partial Differential Equations (PDEs) or Systems of Partial Differential Equations (PDEs). COMSOL 6.2 software provides powerful functionality and flexibility in corrosion simulation, supporting the modelling of a wide range of electrochemical reactions, such as electrode reaction, electrolyte transfer, and diffusion. By building a variety of models and using them in combination, it can simulate the corrosion of metals in the oceanic thermocline environment.

Q345 steel, as a low alloy structural steel, occupies an important position in key areas such as construction, bridges, and especially offshore wind turbine towers due to its excellent physical and mechanical properties and chemical stability. Its high strength, good weldability, and weather resistance make it an ideal choice for offshore wind tower construction. Since offshore wind turbine towers must serve for long periods of time in the special environment of the ocean thermocline, Q345 steel is an important material in this environment. It has good weldability compared to similar materials previously studied, while Q345 steel ensures high strength while also possessing good toughness to effectively resist dynamic loading and fatigue damage in the marine environment.

In the marine environment, the layer in which the seawater temperature changes drastically or discontinuously with depth is called a marine thermocline [21], which is located at water depths of approximately 30–200 m, and its distribution depth, intensity, and thickness change seasonally [22,23]. Marine thermoclines are characterized by high hydrostatic pressures and sharp changes in dissolved oxygen (DO), hydrogen ion (pH), and nutrient concentrations [24,25,26]. Global scholars have carried out many studies on metal corrosion in the submerged zone of seawater (Table 1). Dobson [27] studied the corrosion mechanism of nickel–aluminium bronze in the submerged zone of seawater, Andrade [28] investigated the corrosion rates and mechanisms of concrete reinforcing bars in the submerged zone of seawater, and Gloria [29] studied the localized corrosion characteristics of 2219-T42 and 6061-T6 aluminium alloys in the submerged zone of seawater. However, due to the influences of rapidly changing DO, water temperature, salinity, pH, and dissolved inorganic nitrogen in the marine thermocline [30,31,32,33,34,35], the corrosion of metals is very complicated; at the same time, since the marine thermocline is generally located at water depths of about 30–200 m, it is more difficult to conduct real-sea experiments in this environment. Therefore, few studies have been performed on the corrosion of metals in this specific environment. In this study, the corrosion behaviour and mechanism of Q345 steel in a simulated marine thermocline are investigated via COMSOL numerical simulations and indoor simulation tests. The indoor simulation test [36] is based on the formation mechanism of the marine thermocline, through the design and fabrication of the Marine Thermocline Simulator (MTS), which is used to conduct indoor simulation tests to study the corrosion of Q345 steel located in the marine thermocline. We provide the theoretical basis and data support for the design and formulation of anti-corrosion standards for Q345 steel marine engineering equipment.

## 2. Experiments

### 2.1. Establishment of a Numerical Simulation Model for the Corrosion Behaviour of Q345 Steel

#### 2.1.1. Corrosion Control Equations

According to the law of conservation of matter, the mass balance of the individual substances in the electrolyte solution in this model can be expressed as follows:

(1) $$\frac{{\partial C}_{i}}{\partial t}+\nabla N_{i}=R_{i}$$

where *C_i_* is the concentration of substance *i*, *t* is time, and *N_i_* is the total flux of substance *i*, including the diffusion, electromigration, and convection terms.

The total transport flux $N_{i}$ (mol · m^−2^ · s^−1^) of a very small square cell in a corrosive electrolyte solution satisfies the Nernst–Planck [37] equation, assuming that the charged particles pass through the microcell from the three directions x, y, and z, as shown in Equation (2).

(2) $$N_{i}=-D_{i}\nabla c_{i}-z_{i}Fu_{i}c_{i}\nabla \emptyset_{1}+c_{i}u$$

where $D_{i}$ is the diffusion coefficient of the i charged particle, and the diffusion coefficients of ions in solution involved in this paper, in addition to other parameters, are given in Table 2.

$c_{i}$ is the particle concentration, mol/m^3^.
$z_{i}$ is the charge number.
F is the Faraday constant, F= 96,485 C/mol.
$\emptyset_{1}$ is the electrolyte solution potential, V.
$\nabla \emptyset_{1}$ is the potential difference.
u is the solution flow velocity, m/s.

Since the electrolyte solution is assumed to be stationary in this model and no flow occurs, i.e., the convection term is not considered, $u_{i}$ is the mobility mol·s/kg, which can be obtained from the Nernst–Einstein relationship:

(3) $$u_{i}=\frac{D_{i}}{RT}$$

In this study, with the help of Faraday’s law and Ohm’s law, the movement of charged particles through diffusion and electromigration in an electrolyte solution produces a current, where $i_{1}$(A/m^2^) is the current density vector in the corrosive medium:

(4) $$i_{1}=F\Sigma_{i=1}^{n}Z_{i}(-D_{i}\nabla c_{i}-z_{i}Fu_{i}c_{i}\nabla \emptyset_{1})$$

In the corrosion system modelled in this study, only the cathode and anode with different electrochemical properties on the surface of Q345 steel are relied upon to drive corrosion, without considering the inflow of external current and the application of external potential; therefore, according to Gauss’s law, the current density gradient in the electrolyte solution is 0, and $\nabla i_{1}=0$.

Through the relationship between the potential and charge on the left and right sides of one of the small cells combined with Equations (1)–(3), a typical Laplace equation is obtained to describe the potential distribution characteristics in the corrosive electric field, as shown in Equation (5).

(5) $$\nabla^{2}\emptyset_{1}=\frac{\partial^{2}\emptyset_{1}}{\partial x^{2}}+\frac{\partial^{2}\emptyset_{1}}{\partial y^{2}}+\frac{\partial^{2}\emptyset_{1}}{\partial z^{2}}$$

where $\nabla^{2}$ is the Laplace operator. Numerical simulations are carried out based on the above theory and the boundary conditions obtained from polarization curve measurements.

In this study, when simulating the corrosion behaviours of metals in the simulated environment of the marine thermocline, the following assumptions are made for the electrolyte environment to further simplify the calculations when using COMSOL numerical simulations for the calculations:

(1) The electrolyte solution is a homogeneous medium;
(2) The deposited material is not dissolved in the surface layer of the metal;
(3) The electrolyte solution is electrically neutral.

Therefore, Equation (5) can be simplified to the following expression:

(6) $$\nabla^{2}\emptyset_{1}=0$$

#### 2.1.2. Determination of the Corrosion Boundary Conditions

The polarization behaviour obtained experimentally from the kinetic potential polarization curves was used as the boundary condition. The anode boundary condition is given by Equation (7):

(7) $$\nabla_{n}\phi =-\frac{i_{a}(\emptyset )}{\sigma }$$

The cathode boundary condition can be calculated by Equation (8):

(8) $$\nabla_{n}\phi =-\frac{i_{c}(\emptyset )}{\sigma }$$

where *σ* is the conductivity of the electrolyte solution, $i_{a}(\emptyset )$ is the anodic current density, and $i_{c}(\emptyset )$ is the cathodic current density.

By solving the Laplace equation at the above boundaries, the potential difference and current density distribution in each region can be obtained, and Faraday’s law can be utilized to calculate the corrosion rate in the anodic region [40], as shown in Equation (9):

(9) $$C_{R}=\frac{M}{zF\rho }i_{1}$$

where *F* is the Faraday constant, *M* is the average molar mass, z is the charge transfer number, ρ is the density of the anode carbon steel Q345, and $i_{1}$ is the current density.

**Figure 1:**
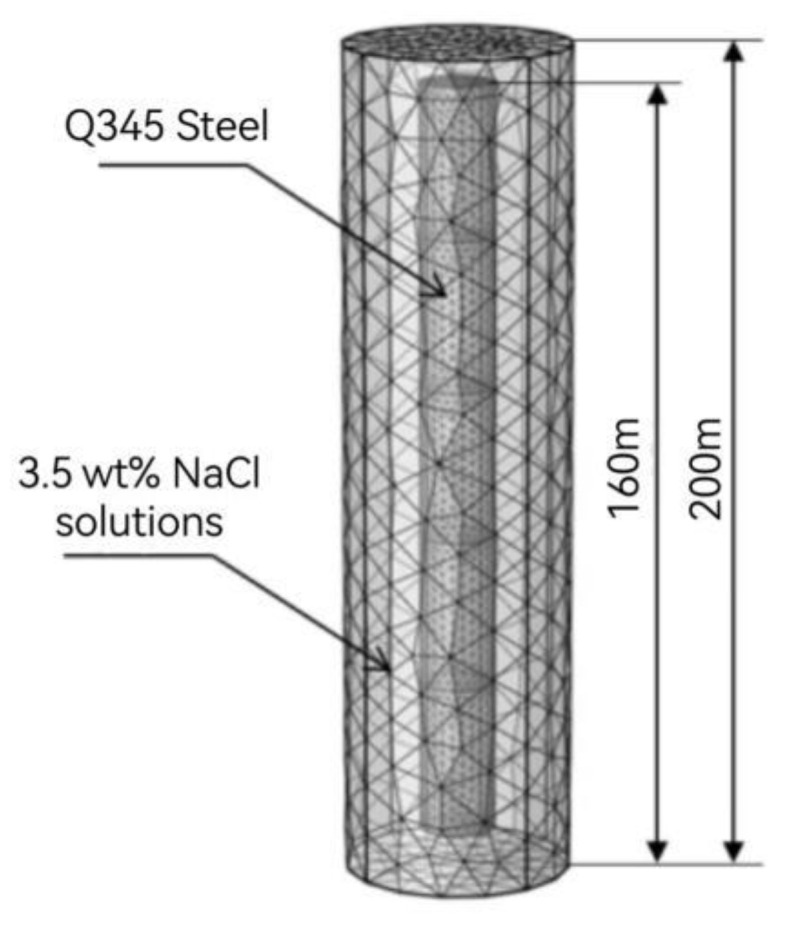
Numerical simulation meshing model of Q345 steel in the marine thermocline simulation environment.

The galvanic coupling corrosion in various regions of Q345 steel after 21 day of immersion in a marine thermocline is simulated in a transient study (Figure 1).

### 2.2. Marine Thermocline Simulation Environment Q345 Steel Corrosion Immersion Experiment

#### 2.2.1. Marine Thermocline Simulator

**Figure 2:**
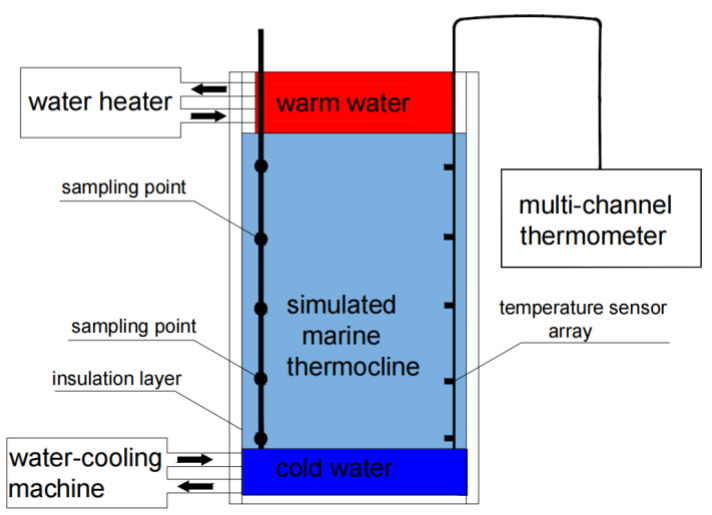
Schematic diagram of the marine thermocline simulation environment test system. The arrow indicates the direction of water flow in the chiller. Color is the difference in water temperature.

The marine thermocline simulator (MTS) consists of three main parts, namely, a holding tank, a specimen rack, and a temperature control system [36]. Given the limitations of the indoor experimental environment, the depth of the oceanic thermocline was simulated at a reduced scale of 1:100. The total depth of the MTS is 200 cm, which is 100 times smaller than that of the actual marine thermocline (i.e., the maximum depth represents an ocean depth of 200 m), as shown in Figure 2. The temperature control system consists of a hot-water heater, a cold-water heater, two thermostatic water tanks, and a set of multiplexed thermometers. A hot-water tank is fixed on top of the simulator and a cold-water tank is fixed on the bottom of the simulator. The water temperature of the hot-water tank is controlled by a circulating industrial hot water machine, and that of the cold-water tank is controlled by a circulating industrial chiller. The seawater at the top of the simulator should be at a higher temperature than that at the bottom, which should be relatively low. The seawater temperature at each location should be measured regularly by a multichannel thermometer. Because of the temperature difference between the top and bottom and the ageing process that is conducted for a certain period, conditions similar to those of a real marine thermocline can be created in the simulation.

#### 2.2.2. Preparation of RCWBE

The experimental material was Q345 steel, and its chemical composition is shown in Table 3. The specimen used for the immersion test had a size of Φ40 mm × 4 mm and was sanded to 1000 mm by water-abrasive sandpaper, and the surface of the specimen was cleaned using acetone and anhydrous ethanol.

**Figure 3:**
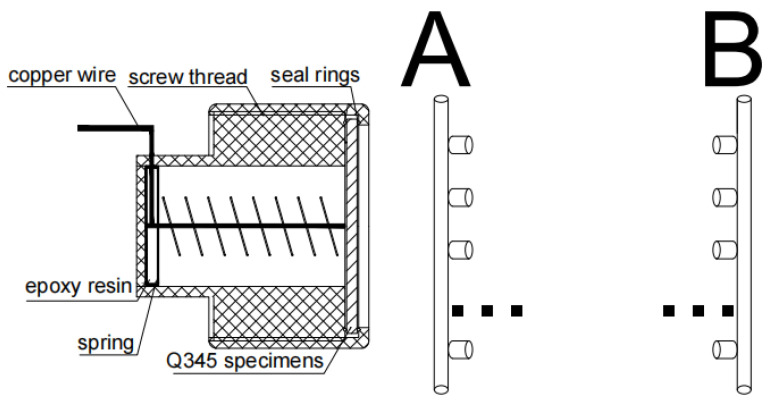
Schematic of RCWBEs. (**A**) Electrochemical measurements and (**B**) morphological observations. There are 5 single electrodes, and ··· is the ellipsis of the simplified diagrams.

In this study, a removable chain wire bundle electrode (RCWBE) was used for modelling Q345 steel in the MTS. The total length of the RCWBE was 160 cm, and the RCWBE consisted of several individual electrodes spaced 40 cm apart from each other. The individual electrodes were made of Q345 steel specimens, rubber rings, PVC screws, and wires, which were sealed with epoxy resin. Figure 3A,B show two different applications of the RCWBE: for electrochemical measurements (A) and for morphological observations (B).

### 2.3. Electrochemical Measurements

To ensure the accurate positioning of each single electrode in the RCWBEs in the MTS, we had to align them vertically at depths of −30 cm, −70 cm, −110 cm, −150 cm, and −190 cm, which were selected based on specific requirements. In situ measurements of the electrochemical noise (ECN) and LP of a type A RCWBE after 21 days of immersion were collected.

#### 2.3.1. Galvanic Corrosion Measurements

In this study, electrochemical noise (ECN) measurements of RCWBEs were carried out to determine the galvanic coupling currents through a CORREST CS350 electrochemical workstation and a three-electrode system. The three-electrode system consisted of two working electrodes (W1 and W2) and one reference electrode (saturated calomel electrode (SCE)). One electrode in each RCWBE was connected to W1, and the other electrodes were simultaneously connected to W2. Electrochemical noise measurements were performed on the entire RCWBE to obtain the galvanic coupling current of each electrode.

#### 2.3.2. Measurement of Instantaneous Icorr

In this study, LP measurements were performed on each electrode to be tested in the RCWBE using a three-electrode system, where the single electrode was considered the working electrode, the 2 × 2 cm^2^ Pt plate was considered the counter electrode, and the SCE was considered the reference electrode. The LP curves were measured by potential scanning in the range of −10~+10 mV at a rate of 0.5 mV/s at the open-circuit potential. The LP data were analyzed to obtain the instantaneous value of Icorr for each electrode.

### 2.4. Corrosion Morphology Analysis

Type B RCWBEs were removed from the MTS after a 21-day immersion test, and the single electrodes at each position were observed by a HITACHI TM4000 Plus (HITACHI High-Tech Corporation, Tokyo, Japan) scanning electron microscope. The SEM was initiated in a vacuum environment with an accelerating voltage of 5 kV, a parameter chosen based on precise consideration of the sample material properties and the expected observation details, aimed at obtaining the clearest image quality without destroying the sample. The fine scanning of the SEM was used to observe details such as tiny cracks, corrosion pits, and corrosion products on the surface of each electrode on the B-type RCWBE one by one.

## 3. Results and Discussion

### 3.1. Analysis of Environmental Factors for Marine Thermocline Simulation

**Figure 4:**
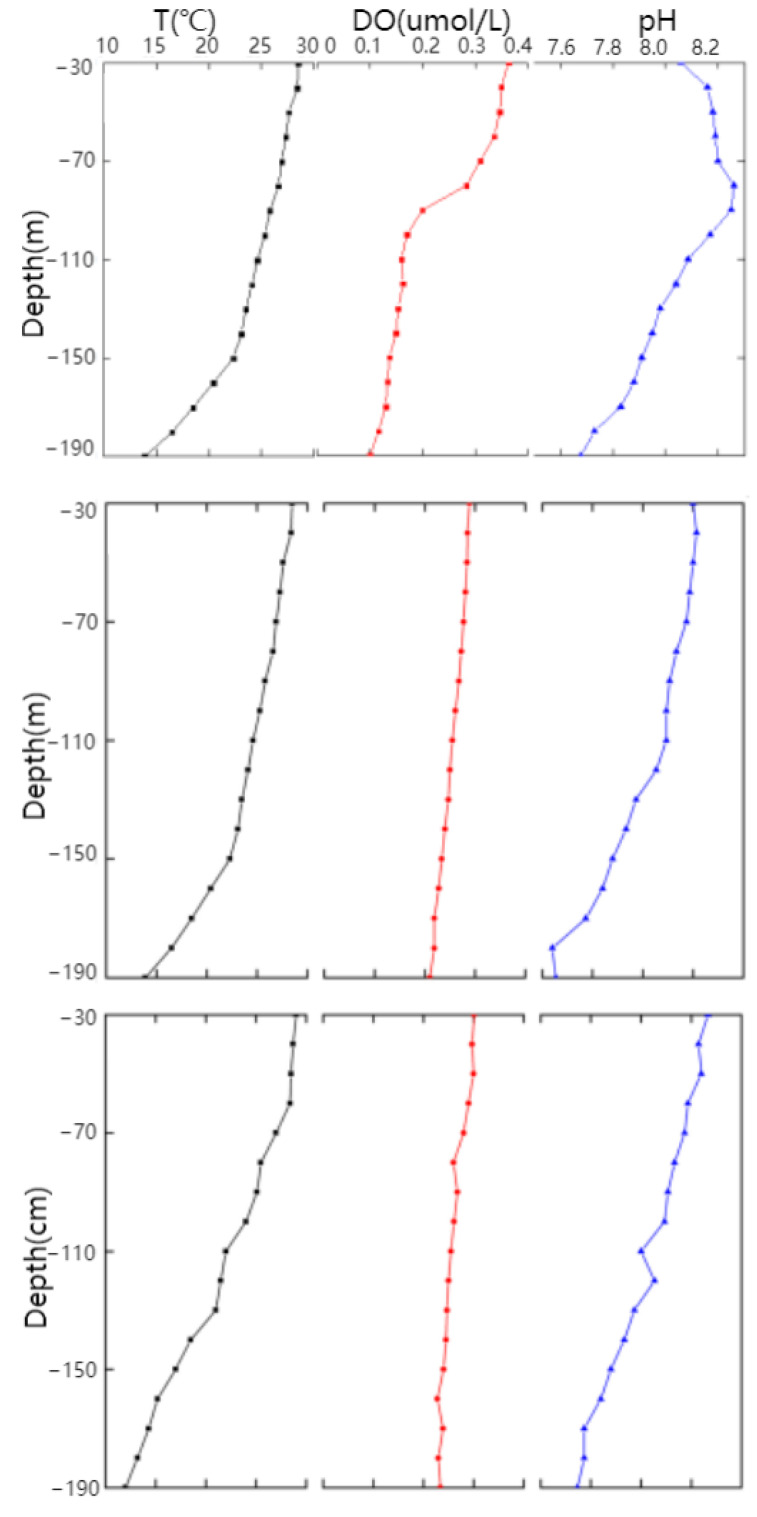
Environmental factors for simulating marine thermocline: natural marine thermocline environment; numerical simulation test; indoor simulation of marine thermocline test.

In this study, marine thermoclines were simulated by COMSOL numerical simulation and an indoor immersion experiment. The total depth of the actual marine thermocline according to the numerical simulation was 200 m, and the total depth of the simulated marine thermocline according to the indoor immersion experiment was 200 cm. The simulated marine thermocline depth was 100 times smaller than the actual depth. Specifically, the maximum depth of 200 cm was representative of the actual depth of 200 m in the marine thermocline. Figure 4 shows the environmental factors of the marine thermocline after 21 d in the numerical simulation and in the indoor immersion experiment. The temperature of the upper layer of seawater is 28 °C, the DO concentration is 0.295 mg/L, and the pH is 8.15. The temperature of the lower layer of seawater is 13 °C, the DO concentration is 0.206 mg/L, and the pH is 7.76. The temperature, DO, and pH of the lower layer of seawater decrease with increasing seawater depth. Moreover, the temperature, DO, and pH of the seawater in the simulated marine thermocline established by the indoor immersion test decrease with increasing seawater depth. The temperature of the upper seawater is 29 °C, the DO concentration is 0.301 mg/L, and the pH is 8.2. The temperature of the lower seawater is 12 °C, the DO concentration is 0.234 mg/L, and the pH is 7.81. By comparing the data of the two types of simulated marine thermoclines, we can see that the temperature of the seawater decreases with increasing seawater depth. According to marine thermocline data, the environmental depths of the two zones differ by a factor of 100, but their environmental factors are basically the same. Therefore, the conclusions drawn from the analysis of the corrosion behaviours of Q345 steels in marine thermoclines established using numerical simulation techniques and indoor immersion experiments should be highly correlated.

### 3.2. Numerical Simulation Experiment Results and Analysis

This study used COMSOL software to numerically simulate the corrosion of Q345 steel in the marine thermocline environment. The polarization behaviour was obtained through the use of polarization curve experiments as boundary conditions for model establishment. After calculation, the corrosion data of Q345 steel in a simulated marine thermocline environment can be obtained.

**Figure 5:**
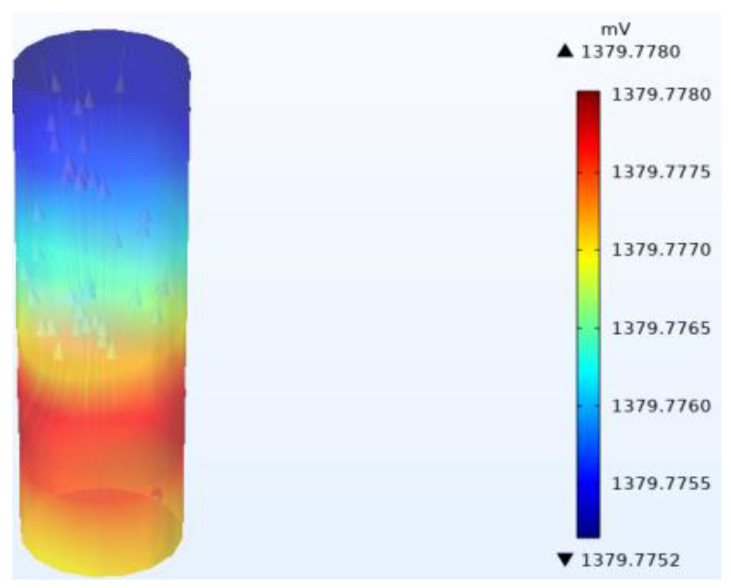
Simulation results of corrosion potential distribution for numerical simulation of Q345 steel in marine thermocline environment. The triangle represents the higher potential part pointing towards the lower potential position.

The galvanic coupling corrosion of Q345 steel in each region after 21 d of immersion in the marine thermocline simulation environment is shown in Figure 5. The maximum corrosion potential region of Q345 steel in the numerical simulation occurs at 150 m, and the minimum corrosion potential region occurs at the top.

**Figure 6:**
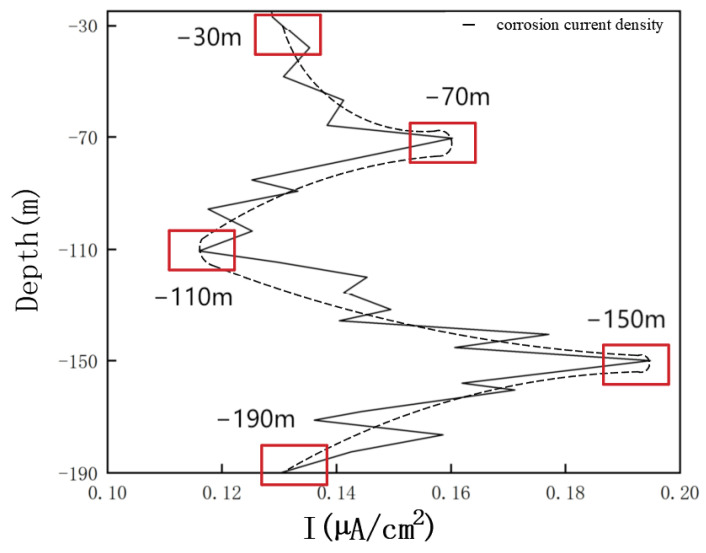
Simulation results of corrosion current density for numerical simulation of Q345 steel in marine thermocline environment. The function of the red square is to highlight the five locations that are of particular concern in this study.

Figure 6 shows the variation curve of the corrosion current density of Q345 steel in the simulated environment of the marine thermocline. It can be concluded that the minimum corrosion current density of Q345 steel is 0.115 µA/cm^2^ at 110 m, and the maximum value is 0.19481 µA/cm^2^ at 150 m. These values indicate that galvanic corrosion of Q345 steel occurs at different depths. The current densities at 70 m and 150 m are obviously greater than those at other locations, which indicates that these two locations act as anodes in the galvanic corrosion reaction. Therefore, the metal corrosion occurring at these locations is the most severe. The current densities at 110 m and 190 m are significantly lower than those at the other locations, indicating that these two locations act as the cathode in the galvanic corrosion reaction and are protected by the anode.

### 3.3. Results and Analysis of the Corrosion Immersion Experiment of Q345 Steel in an Indoor Marine Thermocline Simulation Environment

#### 3.3.1. Analysis of the Galvanic Coupling Corrosion of Q345 Steel in a Simulated 

##### Marine Thermocline

**Figure 7:**
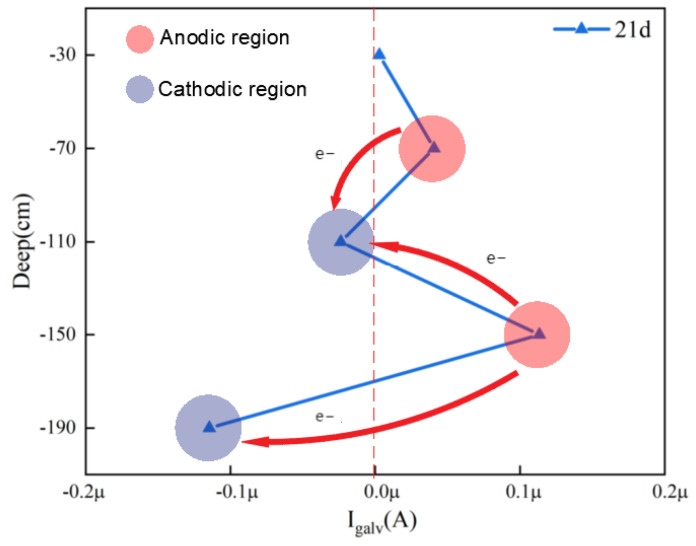
Galvanic coupling currents of Q345 steel in an indoor simulated marine thermocline environment.

Figure 7 shows an analysis of the galvanic current between the electrodes of Q345 steel in the simulated marine thermocline. In the immersion test at 21 d, at depths of 70 cm and 150 cm, where the WBE electric coupling current is positive, the steel serves as the anode in the galvanic corrosion reaction. At depths of 110 cm and 190 cm, where the WBE electric coupling current is negative, the steel serves as the cathode in the galvanic corrosion reaction and is protected by the anode; thus, the corrosion rates of WBEs at 110 cm and 190 cm should be lower than those at 70 cm and 150 cm. The reason for this difference is mainly the oxygen concentration effect, in which the dissolved oxygen content of seawater in the indoor simulated marine thermocline decreases with increasing depth, the concentration of dissolved oxygen (DO) in seawater is greater than that at 70 cm compared with that at 110 cm, and the DO in seawater is greater than that at 150 cm compared with that at 190 cm. Since DO acts as a cathodic depolarizing agent, a location with a high DO content is favourable for the oxygen depolarization process. Therefore, the WBEs at 70 cm and 150 cm are anodes, and the WBEs at 110 cm and 190 cm are cathodes. The results obtained based on the analysis of the interelectrode galvanic currents are in agreement with the analytical results of the numerical simulations.

#### 3.3.2. Analysis of the Corrosion Polarization Curves of Q345 Steel in a Marine 

##### Thermocline Simulation Environment

**Figure 8:**
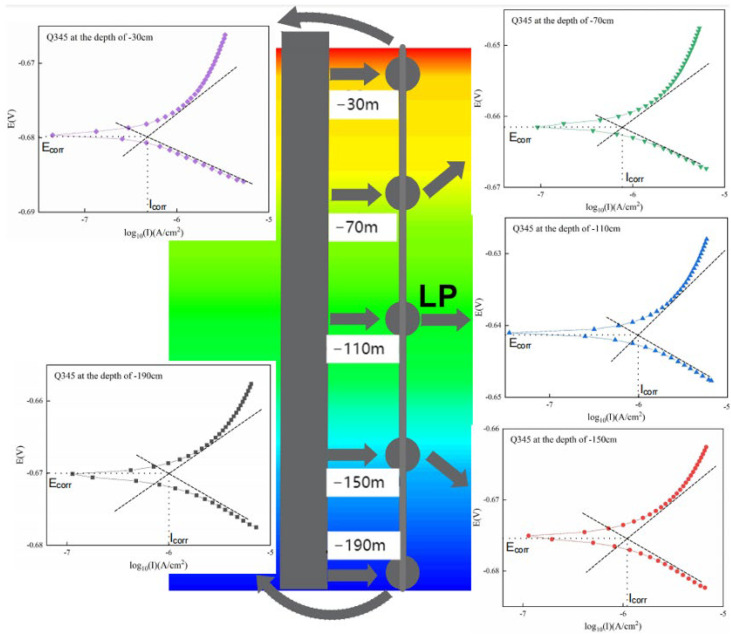
Polarization curve of Q345 steel corrosion in simulated indoor marine thermocline environment. The colors represent the temperatures at different locations in MST, with red indicating the highest temperature, followed by yellow, green ranking third, and blue indicating the lowest temperature.

**Figure 9:**
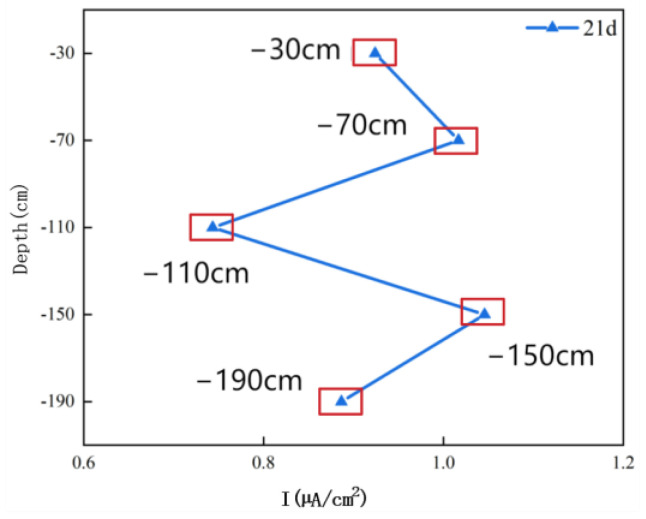
Corrosion current density of Q345 steel in an indoor marine thermocline simulated environment. The function of the red square is to highlight the five locations that are of particular concern in this study.

The polarization curves of Q345 steel corroded in the simulated environment of the marine thermocline are shown in Figure 8, and the results of its corrosion current density fitting are shown in Figure 9. The corrosion current densities of the WBEs at depths of 110 cm and 190 cm are small when they are immersed for up to 21 days, which proves that the corrosion rates of the WBEs at these locations are low. The corrosion current density of the WBE at 150 cm is the largest, followed by that at 70 cm. At these locations, the corrosion current densities are significantly greater than those at other locations. The main reason for this phenomenon is the dissolved oxygen (DO) in seawater and temperature. Dissolved oxygen acts as a cathodic depolarizing agent, and a high dissolved oxygen content is conducive to the oxygen depolarization process. However, an increase in temperature increases the reaction rate and reduces the activation energy of the corrosion reaction, thus accelerating the reaction. Since the dissolved oxygen (DO) concentration and temperature in seawater are greater at 70 cm than at 110 cm, at 150 cm, the DO and temperature in seawater are greater than at 190 cm. This difference leads to the significantly greater corrosion rates of the WBEs at 70 cm and 150 cm than at 110 cm and 190 cm.

#### 3.3.3. Analysis of the Corrosion Morphology of Q345 Steel in a Marine Thermocline 

##### Simulation Environment

**Figure 10:**
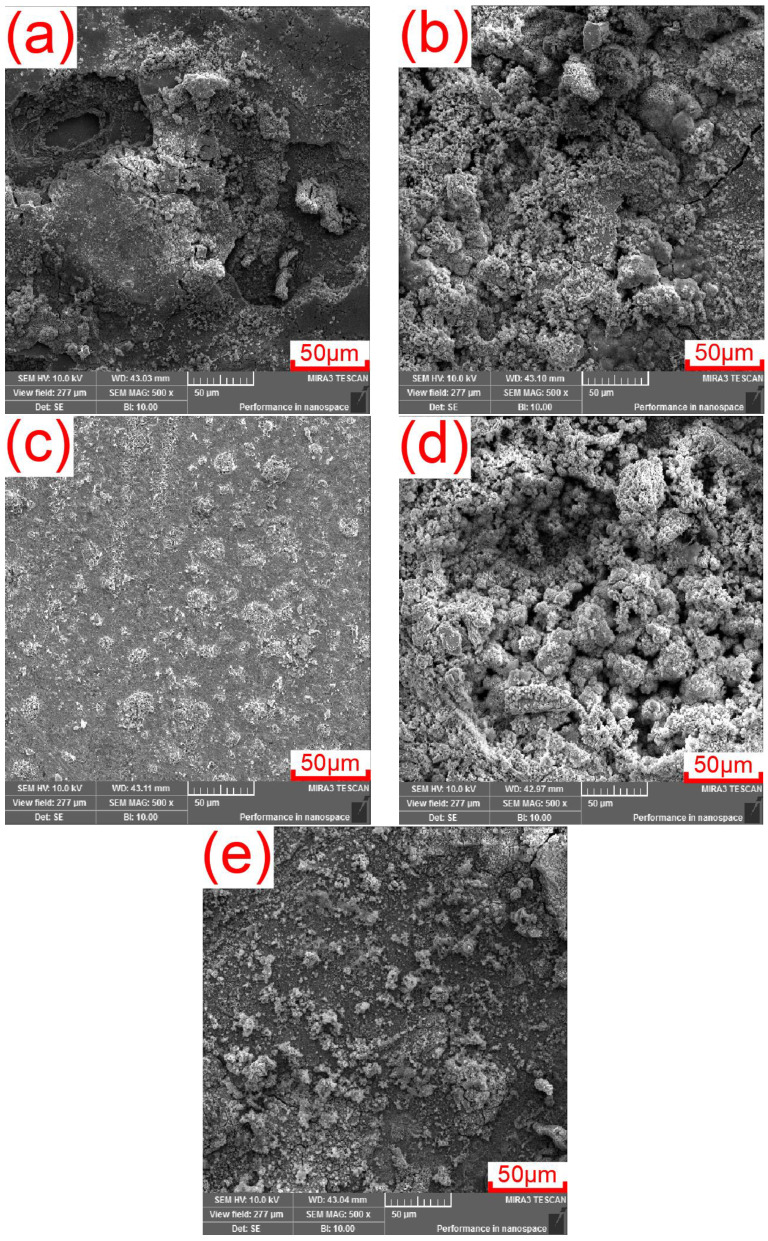
Microscopic corrosion morphology of Q345 steel in indoor simulated marine thermocline environment. Depth of 30 cm (**a**), 70 cm (**b**), 110 cm (**c**), 150 cm (**d**), and 190 cm (**e**).

Figure 10 shows the microscopic corrosion morphologies of WBEs on Q345 steel specimens immersed in a simulated marine thermocline for 21 days. The corrosion products on the surfaces of the WBEs at depths of 110 cm and 190 cm are significantly less abundant than those at other locations. Moreover, the rust layer structure is dense and has good adhesion. The main reason for this is that the electrodes at the above two locations are protected by the anode as cathodes in the electrochemical corrosion reaction. At depths of 70 cm and 150 cm, the amounts of corrosion products on the surfaces of the two electrodes are significantly greater than those at other positions. In addition, the rust layer structure is loose with poor adhesion, and chloride ions easily penetrate the substrate surface, resulting in an accelerated corrosion rate. The results obtained from the corrosion morphology analysis are consistent with those obtained from the numerical simulation analysis.

## 4. Conclusions

The number of metal structures which pass through marine thermoclines is increasing. Therefore, metals must be able to operate in thermoclines for long times to extract offshore oil and gas. In this article, the corrosion behaviours and mechanisms of Q345 steel specimens in marine thermoclines were investigated through COMSOL numerical simulation and a self-developed marine thermocline simulation testing system. The following conclusions were drawn:

(1) A corrosion model of Q345 steel in a marine thermocline was established using COMSOL software. Moreover, certain corrosion data, such as the galvanic current and corrosion current density of Q345 steel after 21 days of immersion were obtained through indoor immersion tests. The results of both methods indicated that galvanic corrosion occurred in Q345 steels at different depths in the simulated marine thermocline.
(2) Through the analysis of the numerical simulation results, it was observed that the Q345 steel specimens at depths of 70 m and 150 m became anodes for galvanic corrosion with high corrosion rates. Conversely, the Q345 steel specimens at depths of 130 m and 190 m became cathodes for galvanic corrosion that were protected by the anode with low corrosion rates.
(3) The main reasons for the galvanic corrosion of Q345 steels are the significant differences in the dissolved oxygen contents and temperatures at different depths in the thermocline. The differences in the dissolved oxygen contents lead to the formation of oxygen concentration potentials in Q345 steels at different depths, thereby triggering galvanic corrosion. Moreover, the temperature difference further exacerbates the degree of galvanic corrosion.
(4) By comparing the results of the numerical simulation experiments and indoor immersion experiments, it can be concluded that the two methods are highly consistent. This observation verifies the reliability of the numerical simulation methods for simulating marine thermoclines and lays the foundation for subsequent research and analysis.

## Figures and Tables

**Table 1:** Studies on metal corrosion in different marine corrosion environments.

Marine atmospheric area	Zhao, Q.Y.; Guo, C.; Niu, K.K.; et. al. [3]	Study of atmospheric corrosion behaviour and mechanism of a 7A85 aluminium alloy.
		The mechanical properties of bare 75A2 alloy degrade significantly in marine air due to a thin electrolyte film on its surface, accelerating corrosion.
Marine tidal range area	Mao, Y.C.; Zhu, Y.; Deng, C.M.; et. al. [4]	Analysis of localized corrosion mechanism of 2024 aluminium alloy at a simulated marine splash zone.
		Metal corrosion rates peak in the marine tidal range area, with notable waterline corrosion, indicating the harshest corrosion environment.
	Fozan, S.A.; Malik, A.U. [5]	Effect of seawater level on corrosion behaviour of different alloys.
		Metal corrosion rates peak in the marine tidal range area, with notable waterline corrosion, indicating the harshest corrosion environment.
	Zhou, X.B.; Wang, Q.; Su, H.; et. al. [7]	Accelerated tidal corrosion of X80 pipeline steel by Desulfovibrio desulfuricans.
		Metal corrosion rates are highest in marine tidal range areas.
Marine total immersion area	Jeffrey, R.; Melchers, R.E. [8]	Corrosion of vertical mild steel strips in seawater.
		The experimental results showed that the intensity of metal corrosion in the submerged zone depends on the length of the sample below the water line to a certain extent, and the longer the sample is, the greater the attenuation.
	Dobson, T.; Larrosa, N.; Reid, M.; et. al. [27]	Corrosion mechanisms of plasma-welded nickel aluminium bronze immersed in seawater.
		The experimental results reveal the corrosion mechanism of plasma-welded nickel aluminum bronze in seawater.
	Acosta, G.; Veleva, L.; LÓPEZ, J.L.; et. al. [29]	Contrasting initial events of localized corrosion on surfaces of 2219-T42 and 6061-T6 aluminium alloys exposed in Caribbean seawater.
		The experimental results reveal contrasting initial corrosion on 2219-T42 and 6061-T6 aluminium alloys in Caribbean seawater.
	Cai, F.F.; Huang, Y.L.; Xu, Y.; et. al. [9]	Study on hydrogen permeation and stress corrosion cracking behaviours of AISI 4135 high-strength steel with macrofouling adhesion in marine immersion zone.
		In the marine immersion zone, macro-scale adhesion cracks facilitate the initiation and growth of local corrosion pits on high-strength steel under natural corrosion and cathodic protection.

**Table 2:** Parameters used in the model [38,39].

Name	Parameter Values	Descriptor
T	298 K	Temperature
F	$96,485\;\;C\cdot {mol}^{-1}$	Avogadro’s number
R	$8.3145\;\;J\cdot ({mol\cdot K)}^{-1}$	Ideal gas constant
Sigma	5.6 S/m	Conductivity of electrolyte solutions
D_O_2_	$1.98\;\times \;10^{-5}\;{cm}^{2}\cdot s^{-1}$	Diffusion coefficient of dissolved oxygen
D_H^+^	$9.401\;\times \;10^{-5}\;{cm}^{2}\cdot s^{-1}$	Diffusion coefficient of H^+^
rho_Fe(OH)_3_	3.8 g/cm^3^	Density of Fe(OH)_3_
M_Fe(OH)_3_	74.8682 g/mol	Molar mass of Fe(OH)_3_
D_Fe^2+^	$0.719\;\times \;10^{-5}\;{cm}^{2}\cdot s^{-1}$	Diffusion coefficient of Fe^2+^
D_Fe(OH)^+^	$0.75\;\times \;10^{-5}\;{cm}^{2}\cdot s^{-1}$	Diffusion coefficient of Fe(OH)^+^
D_Fe(OH)_2_	$0.78\;\times \;10^{-5}\;{cm}^{2}\cdot s^{-1}$	Diffusion coefficient of Fe(OH)_2_

**Table 3:** Chemical composition of Q345 steel.

Element	C	Si	Mn	P	S	Mo	Cr	Ni	Cu	Al	V	Fe
W (%)	0.017	0.500	1.550	0.030	0.025	0.1	0.3	0.4	0.220	0.015	0.110	Margin

## Data Availability

The raw/processed data required to reproduce these findings cannot be shared at this time due to technical or time limitations. They will be available on request.
